# Main characteristics observed in patients with hematologic diseases admitted to an intensive care unit of a Brazilian university hospital

**DOI:** 10.5935/0103-507X.20150034

**Published:** 2015

**Authors:** Lídia Miranda Barreto, Júlia Pereira Torga, Samuel Viana Coelho, Vandack Nobre

**Affiliations:** 1 Hospital das Clínicas, Universidade Federal de Minas Gerais - Belo Horizonte (MG), Brazil.; 2 Postgraduate Program in Infectious Diseases and Tropical Medicine, Faculdade de Medicina, Universidade Federal de Minas Gerais - Belo Horizonte (MG), Brazil.

**Keywords:** Respiratory insufficiency, Respiration, artificial, Hematologic diseases, Noninvasive ventilation, Treatment outcome, Intensive care units

## Abstract

**Objective:**

To evaluate the clinical characteristics of patients with hematological disease admitted to the intensive care unit and the use of noninvasive mechanical ventilation in a subgroup with respiratory dysfunction.

**Methods:**

A retrospective observational study from September 2011 to January 2014.

**Results:**

Overall, 157 patients were included. The mean age was 45.13 (± 17.2) years and 46.5% of the patients were female. Sixty-seven (48.4%) patients had sepsis, and 90 (57.3%) patients required vasoactive vasopressors. The main cause for admission to the intensive care unit was acute respiratory failure (94.3%). Among the 157 studied patients, 47 (29.9%) were intubated within the first 24 hours, and 38 (24.2%) underwent noninvasive mechanical ventilation. Among the 38 patients who initially received noninvasive mechanical ventilation, 26 (68.4%) were subsequently intubated, and 12 (31.6%) responded to this mode of ventilation. Patients who failed to respond to noninvasive mechanical ventilation had higher intensive care unit mortality (66.7% versus 16.7%; p = 0.004) and a longer stay in the intensive care unit (9.6 days versus 4.6 days, p = 0.02) compared with the successful cases. Baseline severity scores (SOFA and SAPS 3) and the total leukocyte count were not significantly different between these two subgroups. In a multivariate logistic regression model including the 157 patients, intubation at any time during the stay in the intensive care unit and SAPS 3 were independently associated with intensive care unit mortality, while using noninvasive mechanical ventilation was not.

**Conclusion:**

In this retrospective study with severely ill hematologic patients, those who underwent noninvasive mechanical ventilation at admission and failed to respond to it presented elevated intensive care unit mortality. However, only intubation during the intensive care unit stay was independently associated with a poor outcome. Further studies are needed to define predictors of noninvasive mechanical ventilation failure.

## Abstract (pt)

**Objetivo:**

Avaliar as características clínicas de pacientes com doenças hematológicas admitidos à unidade de terapia intensiva e o uso de ventilação mecânica não invasiva em um subgrupo com disfunção respiratória.

**Métodos:**

Foi realizado um estudo retrospectivo observacional em pacientes admitidos entre setembro de 2011 e janeiro de 2014.

**Resultados:**

Foi incluído um total de 157 pacientes. A média de idade foi de 45,13 (± 17,2) anos, sendo que 46,5% dos pacientes eram do sexo feminino. Sessenta e sete (48,4%) dos pacientes tinham sepse e, em 90 (57,3%) pacientes, foi necessária a utilização de vasopressores. A principal razão para admissão à unidade de terapia intensiva foi a insuficiência respiratória aguda (94,3%). Dentre os 157 pacientes avaliados, 47 (29,9%) foram intubados nas primeiras 24 horas, e 38 (24,2%) foram submetidos à ventilação mecânica não invasiva. Dentre os 38 pacientes que receberam inicialmente ventilação não invasiva, 26 (68,4%) foram subsequentemente intubados, e 12 (31,6%) responderam a essa modalidade ventilatória. Pacientes que deixaram de responder à ventilação mecânica não invasiva tiveram maior mortalidade na unidade de terapia intensiva (66,7% *versus* 16,7%; p = 0,004) e um tempo maior de permanência na unidade de terapia intensiva (9,6 dias *versus* 4,6 dias; p = 0,02), quando comparados aos casos em que se obteve sucesso com a ventilação mecânica não invasiva. Os escores basais de gravidade (SOFA e SAPS 3) e a contagem total de leucócitos não foram significantemente diferentes entre esses dois grupos. Em um modelo de regressão logística multivariada que incluiu os 157 pacientes, intubação a qualquer momento durante a permanência na unidade de terapia intensiva e SAPS 3 associaram-se de forma independente com mortalidade na unidade de terapia intensiva, enquanto o uso de ventilação mecânica não invasiva não apresentou essa correlação.

**Conclusão:**

Neste estudo retrospectivo com pacientes hematológicos graves, aqueles submetidos a ventilação mecânica não invasiva quando da admissão e que tiveram falha da resposta tiveram uma alta mortalidade na unidade de terapia intensiva. Entretanto, apenas intubação durante a permanência na unidade de terapia intensiva se associou de forma independente com desfechos indesejáveis. São necessários mais estudos para definir preditores da falha da ventilação mecânica não invasiva.

## INTRODUCTION

The number of patients with hematologic malignancies admitted to intensive care units (ICUs) has grown in recent years. This increase is largely due to the greater awareness of these diseases coupled with specific therapeutic advances made in recent decades, which have further justified the transfer of severely ill onco-hematologic patients to the ICU.^(1-4)^

However, despite the advances observed in recent years, the mortality of hematological patients in the ICU is still high - between 50 and 70% - especially among those who require invasive mechanical ventilation (IMV) or suffer two or more organ failures.^(2)^ The most common acute conditions in these populations arise from underlying diseases (e.g., hyperleukocytosis) and treatment-related complications.^(1)^

Acute respiratory failure represents the major organ dysfunction in patients with hematologic malignancies admitted to the ICU.^(1,5)^ According to certain reports, the early and well-indicated use of noninvasive mechanical ventilation (NIMV) in these patients is associated with a reduced need for IMV and intubation upon admission, reduced complications during hospitalization, and reduced mortality in the ICU and in the hospital.^(6-8)^ Among patients submitted to NIMV trials, those requiring subsequent IMV have worse outcome compared to successful cases. Therefore, failure of NIMV is associated with a higher complication rate, a longer duration of IMV, a longer stay in the ICU and a higher mortality rate.^(9)^ Therefore, a better understanding of the predictors of success with this ventilator strategy is strongly required. Data regarding the characteristics of hematologic patients admitted to ICU in Brazil are limited.

In the present study, we evaluated a population of critically ill patients with hematologic diseases, notably malignancies, admitted to an 18-bed mixed ICU in Brazil. We aimed to investigate the use of different modalities of ventilatory support in this population (e.g., IMV and NIMV) and its consequences, as well as examine aspects associated with the patients’ outcomes.

## METHODS

A retrospective observational study was performed, including adult patients admitted to the ICU from September 2011 to January 2014. This study was approved by the local Research Ethics Committee (CAAE - 37297314.5.0000.5149). Because of its retrospective nature, the requirement of obtaining informed consent from participating patients was waived.

All subjects aged ≥ 18 years with hematologic diseases and admitted to the intensive care unit of the *Hospital das Clínicas* of *Universidade Federal de Minas Gerais* (HC-UFMG) during the period of interest were included in the study. The HC-UFMG is a 506-bed university hospital located in southeastern Brazil that is intended for high complexity care.

Clinical and laboratory information was obtained by consulting a prospectively filled database (*Epimedmonitor.com*). During the period considered (27 months), 754 patients were admitted to the ICU and, among them, 157 (20.8%) suffered from hematologic diseases.

Hematologic diagnoses had been previously defined or were determined upon admission by the hematology and intensive care teams. The reasons for admission to the ICU were defined by the intensive care team through the analysis of signs and symptoms, as well as laboratory exams and imaging.

In addition to demographic data (gender and age), the following variables were analyzed: hematologic diagnosis, severity scores for the first 24 h after admission to the ICU - Simplified Acute Physiology Score 3 (SAPS 3) and Sequential Organ Failure Assessment (SOFA), presence of severe comorbidities, use of antibiotics and antifungals upon admission, total leukocyte count, respiratory rate and blood gas parameters upon admission, need for mechanical ventilation and modalities (IMV or NIMV), use of vasopressors, need for hemodialysis, shock, use of chemotherapy in the ICU, development of acute respiratory distress syndrome, duration of mechanical ventilation and weaning, length of stay in the ICU, limitation of therapeutic effort, and condition of hospital discharge (survival vs. death).

### Definitions

Acute hypoxemic respiratory failure was defined as oxygen saturation below 90% and/or PaO_2_ below 60mmHg on room air, presence of dyspnea and respiratory rate above 30 breaths per minute.^(10)^

Invasive mechanical ventilation was defined as ventilatory support to patients with acute or decompensated chronic respiratory failure by means of an artificial airway (endotracheal tube or tracheotomy). This mode of ventilation was used on patients with respiratory failure who failed to respond to noninvasive support or had no initial indication for it, according to judgment of the intensive care team (e.g., severity of the clinical/respiratory condition). Conventional modes of ventilation were used on patients who underwent invasive appropriate ventilatory support, as indicated by the intensive care team.^(11,12)^

Noninvasive mechanical ventilation was defined as ventilatory support by means of an interface (mask) in a noninvasive manner. The face mask was manually adjusted to the patient’s face with the aid of an adapter, or “harness,” until the patient was harmonically synchronized with and well-adapted to the ventilator. The mode used was pressure support ventilation + continuous positive airway pressure (PSV + CPAP) with baseline CPAP levels set at 5cmH_2_O and pressure support starting at 5cmH_2_O, according to the optimal tidal volume and sensitivity to pressure (1 to 2cmH_2_O) or flow (3 to 5L/s), with FIO_2_ adjusted to maintain SpO_2_ > 92%. Patients were analyzed globally as they successfully avoided intubation or failed to do so, in which case they were intubated after an initial attempt at NIMV.

According to the ICU protocol, the following strategy is adopted in patients under NIMV: serial collection of arterial blood gases, increase of pressure support (2 to 3cmH_2_O at a time) when blood gases are required or respiratory distress occurs, and increase of FIO_2_ and/or CPAP in cases of persistent hypoxemia with SaO_2_ or SpO_2_ < 92% and/or PaO_2_ < 60mmHg. The decision regarding patient intubation was made by the physician in charge and usually occurred when there was aggravation of the underlying disease, a decreased level of consciousness, hemodynamic instability or cardiac arrhythmias, agitation requiring sedation, inability of the patient to clear secretions, worsening blood gas levels unresponsive to noninvasive ventilation, refractory hypoxemia (SpO_2_ < 92% or PaO_2_ < 60mmHg) and risk of imminent cardiac arrest. When NIMV was no longer necessary (with an improvement in respiratory pattern, blood gases and basic chest radiography), ventilation was gradually withdrawn.^(11,12)^

### Statistical analysis

Data obtained from quantitative variables were expressed as the mean (± standard deviation - SD) or median (P25% - P75%), according to the data distribution (normal or non-normal). Comparative analyses for frequency were performed using the chi-square test and Fisher’s exact test when appropriate, and comparative analyses for continuous variables were performed using *Student’s t*-test or the Mann-Whitney U test.

To investigate the influence of NIMV and IMV on hospital mortality, we performed a multivariate analysis using a logistic regression model, built in a forward strategy. Except for age, which was included in an *a priori* faction, only variables that reached p < 0.2 in a univariate analysis for mortality were included in the final model. Goodness-of-fit of the multivariate model was evaluated by the Hosmer and Lemeshow test.

In all analyses, the significance level was set at 5%. The statistical software used was the Statistical Package for the Social Sciences (SPSS), version 19.1.

## RESULTS

Overall, 157 patients were included in the study. Among them, 46.5% were female, and the mean age was 45.13 (± 17.2) years. Most patients (approximately 80% of the cases) suffered from malignant diseases, such as myelodysplasia. The main hematologic diagnoses were acute myeloid leukemia (38.2%), bone marrow aplasia (12.8%) and myelodysplasia (12.5%). The main characteristics presented by patients upon admission are listed in table 1.

**Table 1:** Characteristics of patients upon inclusion in the study (N = 157)

**Characteristic**	**Values**
Age (years)	45.13 ± 17.9
Gender	
Male	84 (53.5)
Female	73 (46.5)
Characteristics upon admission to the ICU	
Acute respiratory failure	77 (49.0)
OI > 200	133 (84.7)
Orotracheal intubation (first 24 hours)	47 (29.9)
Noninvasive mechanical ventilation (first 24 hours)	38 (24.2)
Use of antibiotics	52 (33.1)
SOFA	6.0 (4.0 - 9.5)
SAPS 3	67 (53.5 - 76.0)
Lactate (mmol/L)	1.4 (1.0 - 2.6)
Total leukocytes (cells/mm^3^)	6.0 (0.6 - 18.6)
Respiratory rate (bpm)	26 (21 - 29)
PaO_2_ (mmHg)	103 (81 - 141)
PaO_2_/FIO_2_ (mmHg)	304 (207 - 389)
Hematologic diseases	
Malignant	128 (81.6)
Acute myeloid leukemia	58 (36.9)
Non-Hodgkin lymphoma	15 (9.6)
Acute lymphocytic leukemia	12 (7.7)
Multiple myeloma	9 (5.8)
Myelodysplasia	7 (4.5)
Myeloproliferative neoplasm	7 (4.5)
Hodgkin's lymphoma	6 (3.8)
After autologous BMT	6 (3.8)
After allogeneic BMT	3 (1.9)
Chronic lymphocytic leukemia	3 (1.9)
HTLV-associated T-cell leukemia	1 (0.6)
Myelofibrosis	1 (0.6)
Nonmalignant	29 (18.4)
Bone marrow aplasia	12 (7.6)
Sickle cell anemia	8 (5.1)
Non neoplasic febrile neutropenia	8 (5.1)
Immune thrombocytopenic purpura	1 (0.6)
Main comorbidities	
Cirrhosis	1 (0.6)
COPD	6 (3.8)
Heart failure	5 (3.2)
Chronic renal failure (with dialysis)	2 (1.2)

ICU - intensive care unit; OI - oxygenation index; Bpm - breaths per minute; SOFA - Sequential Organ Failure Assessment; SAPS 3 - Simplified Acute Physiology Score 3; PaO_2_ - arterial oxygen tension; BMT - bone marrow transplantarion; HTLV - human T lymphotropic virus; COPD - chronic obstructive pulmonary disease. Values include: mean, number with percentage and median ± standard deviation.

Around one-third of the patients were admitted while using antibiotics and antifungals - 33.1% and 23.6%, respectively - mainly due to infectious processes in the lungs. The major cause for admission to the ICU was acute respiratory failure. During the first 24 hours following admission, 47 (29.9%) of the 157 patients were intubated and 38 (24.2%) underwent noninvasive mechanical ventilation (Table 1).

Almost half (48.4%) of the patients developed sepsis, and 57.3% required vasoactive vasopressors during their stay in the ICU. Other data regarding baseline characteristics and follow-up data, comparing patients according to their outcome in the ICU, are depicted in table 2.

**Table 2:** Univariate analysis of the main characteristics and outcomes, according to the intensive care unit mortality and including the 157 studied patients

**Characteristics**	**Values**	**Survivor**	**Non-survivor**	**OR**	**95%CI**	**p value**
At the time of ICU admission						
Age	45.13 (± 17.2)	44.06 (± 16.55)	46.31 (± 18.13)	1.009	0.991 - 1.028	0.336
Malignant disease	128 (81.53)	71 (55.47)	57 (44.53)	0.829	0.402 - 1.712	0.613
SAPS 3 values	67 (53.2 - 76.0)	59 (50 - 68)	74 (66.5 - 81)	1.050	1.024 - 1.076	< 0.001
Baseline lactate	1.4 (1.0 - 2.6)	1.3 (0.9 - 2.3)	1.5 (1 - 2.6)	1.119	0.954 - 1.313	0.166
NIMV	38 (24.2)	19 (50)	19 (50)	0.896	0.404 - 1.983	0.786
IMV	47 (29.9)	15 (31.91)	33 (70.21)	2.906	1.423 - 5.934	0.003
PaO_2_/FiO_2_	304 (207 - 389)	315 (233 - 404.5)	296 (182 - 360)	0.997	0.995 - 1.000	0.027
Sepsis	67 (42.68)	34 (50.75)	33 (49.25)	0.787	0.467 - 1.327	0.369
Outcomes during ICU stay						
NIMV	48 (30.6)	25 (52.08)	23 (47.92)	1.330	0.645 - 2.744	0.440
IMV	99 (63.1)	31 (31.31)	68 (68.68)	19.933	7.228 - 50.415	< 0.001
Tracheotomy	17 (10.8)	10 (58.82)	7 (41.18)	0.741	0.267 - 2.058	0.565
Hemodialysis	39 (24.84)	11 (28.21)	28 (71.79)	0.248	0.114 - 0.240	< 0.001
Chemotherapy	20 (12.74)	12 (60)	8 (40)	0.580	0.228 - 1.473	0.252
Use of vasopressors	90 (57.3)	25 (27.77)	65 (72.22)	17.722	7.625 - 41.191	< 0.001
ARDS	19 (12.1)	5 (26.3)	14 (73.7)	4.875	1.539 - 15.445	0.007

OR - odds ratio; 95%CI - 95% confidence interval; ICU - intensive care unit; SAPS 3 - Simplified Acute Physiology Score 3; NIMV - noninvasive mechanical ventilation; IMV - invasive mechanical ventilation; PaO_2_/FiO_2_ - partial pressure of oxygen/fraction of inspired oxygen; ARDS - acute respiratory distress syndrome. Values include: mean, number with percentage and median ± standard deviation.

**Figure 1:**
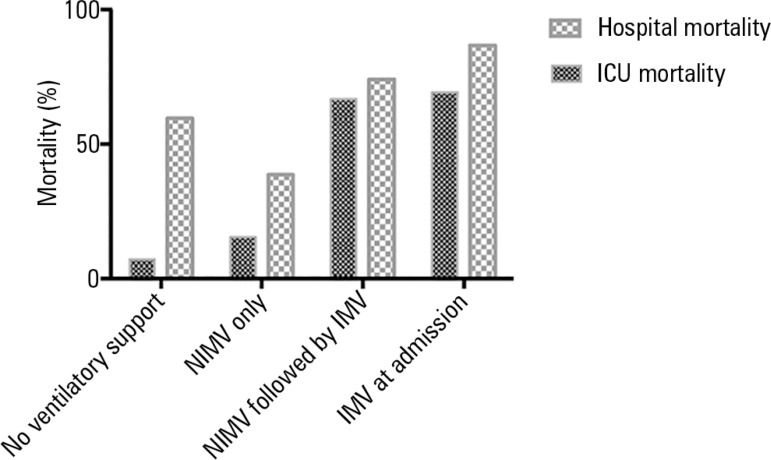
Mortality in the intensive care unit and hospital among 157 hematologic patients according to the ventilatory support received during the intensive care unit stay. ICU - intensive care unit; NIMV - noninvasive mechanical ventilation; IMV - invasive mechanical ventilation.

Among the 38 patients who initially received NIMV, 26 (68.43%) were subsequently intubated and 12 (31.57%) responded favorably to this mode of ventilation. Overall, ICU mortality and hospital mortality were 47.8% and 73.2%, respectively (Figure 1). As shown in figure 1, overall mortality increased from the subgroup of patients with no need for mechanical ventilator support during the ICU stay (lowest mortality, 7.1%) to the those subgroups receiving any type of ventilator support, either non-invasive or invasive (mortality as high as 69.3%).

Specifically for ICU mortality, this endpoint was significantly higher in patients who failed to respond to NIMV compared to those with a good response (66.7% versus 16.7%; p = 0.004). The mortality observed among patients who failed to respond to NIMV and underwent intubation resembled that of the 47 patients who were intubated within the first hours of admission to the ICU (69.3%) with a non-significant difference (p = 0.79). The length of stay in the ICU was higher among patients who were intubated after failing NIMV, when compared with patients with a good initial response (9.0 days versus 4.5 days, p = 0.02).

At baseline, patients who failed NIMV showed lower oxygenation indices (PaO_2_/FiO_2_ < 200 in 92.3% of the cases, compared with 41.7% of the patients with an appropriate response; p = 0.002) and a more frequent use of vasopressors (80.8% versus 8.3%; p = 0.001). However, there was no significant difference between the two groups (NIMV only versus NIMV followed by IMV) with respect to gender, presence of comorbidities, lactate level upon admission and median values for total leukocytes, SOFA and SAPS 3 within the first 24 hours in the ICU (Table 3).

**Table 3:** Characteristics of patients treated with noninvasive mechanical ventilation during the first 24 hours following intensive care unit admission. Patients requiring only noninvasive mechanical ventilation are compared with patients requiring invasive mechanical ventilation as a rescue therapy for noninvasive mechanical ventilation

**Characteristics**	**Noninvasive mechanical ventilation only (N = 12)**	**Invasive mechanical ventilation following noninvasive mechanical ventilation (N = 26)**	**p value**
Age (years)	41 (20)	44 (16)	0.505
Female	7 (58.3)	17 (65.4)	0.72
OI < 200	7 (58.3)	24 (92.3)	0.022
Use of vasopressors	1 (8.3)	21 (80.8)	< 0.001
Stay in the ICU (days)	4.6 (3 - 7)	9.6 (3.75 - 13)	0.02
Mortality in the ICU	2 (16.7)	18 (69.2	0.004
Total leukocytes (cells/mm^3^)	2.070 (402 - 7.545)	7.085 (1.205 - 27.447)	0.137
SAPS 3	65 (51 - 75)	67 (52 - 75)	0.591
SOFA	6 (4.0 - 10.0)	7.5 (5.2 - 10.7)	0.342

OI - oxygenation index; ICU - intensive care unit; SAPS 3 - Simplified Acute Physiology Score 3; SOFA - Sequential Organ Failure Assessment. Values include: mean, number with percentage and median ± standard deviation.

To investigate which variables were associated with hospital mortality in the whole sample of patients, we performed a logistic regression analysis. In addition to age, which was included *a priori*, lactate levels at admission, PaO_2_/FIO_2_ at admission, SAPS 3 values measured within the first 24 hours of the ICU stay and IVM at any point of ICU stay were entered in the model (Table 2). As presented in table 4, from these variables, only SAPS 3 values and IVM during the ICU stay were independently associated with ICU mortality.

**Table 4:** Multivariate analysis of intensive care unit mortality, including the 157 studied patients

**Characteristics**	**OR**	**95%CI**	**p value**
SAPS 3 values	1.042	1.012 - 1.072	0.006
Invasive mechanical ventilation during ICU stay	15.275	5.768 - 40.455	< 0.001

OR - odds ratio; 95%CI - 95% confidence interval; SAPS 3 - Simplified Acute Physiology Score 3; ICU - intensive care unit.

## DISCUSSION

In the present study, we observed that all-cause hospital mortality was high in a group of critically ill patients with hematologic diseases, mainly malignancies, although it did not differ from the rates observed in other populations affected by conditions associated with bad outcomes (e.g., septic shock).^(13)^ Moreover, patients with a good response to NIMV instituted during the first 24 hours following admission to the ICU had a good prognosis. Patients who failed this mode of ventilation and required subsequent orotracheal intubation exhibited highly increased mortality rates, which were above 60%.

In other series published in literature, mortality in patients admitted to ICUs with hematologic diseases, especially malignancies, was also high, exceeding 50%.^(1)^ The increased mortality inherent to the underlying hematologic diseases, coupled with the lack of knowledge on the critical conditions affecting this population, prompted questioning about the benefit of transferring them to the ICU throughout the years. However, recent data available in the literature indicate improved prognoses for these patients, suggesting that mortality depends not only on the prognosis of the hematologic disease but also on the nature of the acute complications.^(2)^ Recent studies that reported better results in the treatment of critically ill hematological patients have led to broader policies for admission to the ICU, in which a good performance status and the availability of treatments to prolong survival are considered to be the main criteria.^(4)^

Among hematologic patients, the major cause for admission to the ICU is acute respiratory failure. In these patients, the incidence of pulmonary infections associated with sepsis is elevated due to immunosuppression (e.g., neutropenia). Acute respiratory failure requiring mechanical ventilation is an important prognostic factor for both mortality and the need for inotropic therapy.^(1)^ There are questions regarding the best mode of ventilatory support for onco-hematologic patients with acute respiratory dysfunction. In a seminal study published in 2001, Hilbert et al. reported the benefit of NIMV in patients with acute respiratory failure and pneumonia who were immunocompromised for different reasons.^(6)^ The initial enthusiasm for this mode of ventilation was countered by the results of some later studies, which demonstrated an increased mortality when NIMV failed and the patient underwent orotracheal intubation.^(14-16)^

Although not consensual,^(17,18)^ specifically in patients with hematologic malignancies, observational data suggested the early use of CPAP (NIMV) is beneficial in increasing survival, reducing the need for endotracheal intubation and IMV, reducing mortality in the hospital and in the ICU, reducing complications and improving blood gas and oxygenation indices.^(8,19,20)^ This benefit could even affect patients with severe hypoxemia,^(16)^ although increased mortality has been described when this mode of ventilation was employed instead of IMV in cases of acute respiratory distress syndrome.^(9)^

On the other hand, in hematological patients, the failure of NIMV is associated with a longer duration of mechanical ventilation, longer stay in the ICU and higher mortality rate, both in the hospital and in the ICU. This fact can be explained by the mode of NIMV employed, by the etiology of the respiratory failure, and by the intrinsic complexity of hematological patients with acute respiratory failure, making it difficult to predict whether NIMV will be successful.^(17,18,21)^ In general populations admitted to the ICU with acute respiratory failure, some predictors of NIMV failure have been described, such as the use of vasopressors, renal replacement therapy, severe hypoxemic respiratory failure, comorbidities, late transfer to the ICU and delay in administering appropriate ventilatory support.^(20,22,23)^ However, there is still no specific consensus regarding onco-hematologic patients.

As found in our multivariate analysis, the need for IMV, whether at admission or later on the ICU stay, is strongly associated with hospital mortality. However, using NIMV at all costs to avoid invasive therapy may be harmful if the need for tracheal intubation is already present. It is reasonable to hypothesize that some individuals initially treated with NIMV would have performed better if they received IMV instead of NIMV as the first ventilatory modality. Defining whether the use of NIVM is safe and potentially beneficial remains a challenging decision and must be conducted in an individual basis.

This study has numerous limitations that must be mentioned. First, this was single-center study, which limits generalization of the data to other settings. Second, even though our main source of data was prospectively filled, the retrospective design of this study may be associated with some incorrect information. Third, although the severity scores (SAPS 3 and SOFA) were not significantly different between the two subgroups undergoing NIMV upon admission to the ICU (success versus failure of NIMV), the small number of patients included in the analysis limited the use of multivariate techniques to verify the independent contribution of the chosen mode of mechanical ventilation on hospital mortality. Finally, even though our focus of interest was patients with hematological neoplasia, almost 20% of patients had no malignant diseases. Unfortunately, we were not able to perform a subgroup analysis excluding the non-neoplastic diagnoses due to the limited sample of patients.

## CONCLUSION

According to our results, critically ill onco-hematologic patients present increased mortality rates, albeit incompatible with any *a priori* restriction of their transfer to intensive care units. Moreover, the choice of noninvasive mechanical ventilation instead of invasive mechanical ventilation in onco-hematologic patients with acute respiratory failure must follow very strict criteria. When the initial choice is made in favor of the noninvasive method, the patient must be reassessed within the next hour to decide whether orotracheal intubation will be necessary. Prospective studies with larger populations are imperative to establish protocols for specific ventilatory strategies in patients with hematologic malignancies admitted to the intensive care unit.
